# Supplementary material for: Chronic pain: Evidence from the national child development study
Source: PLoS One. 2022 Nov 2;17(11):e0275095. doi: 10.1371/journal.pone.0275095 (PMC9629596; doi:10.1371/journal.pone.0275095)
Supplement: S1 Appendix — (DOCX) [file pone.0275095.s001.docx]

Appendix. Pain questions from 2002-2004 Biomedical study
